# Specific treatment for alcohol use disorder reduces relapse after psichiatric hospitalization

**DOI:** 10.1192/j.eurpsy.2023.1189

**Published:** 2023-07-19

**Authors:** O. Martin-Santiago, J. I. Goncalves-Cerejeira, G. Guerra-Valera, M. Calvo-Valcarcel, P. Martinez-Gimeno

**Affiliations:** 1 Hospital Clinico Universitario de Valladolid, Valladolid; 2 Complejo asistencial de Palencia, Palencia, Spain

## Abstract

**Introduction:**

Patients with an alcohol use disorder frequently relapse after various efforts to quit. Admission to hospital units is a possible start to stop drinking alcohol and reach abstinence. Among the pharmacological strategies to quit this addiction are specific drugs, such as disulfiram or namelfen, which are widely studied. Hospitalized patients frequently initiate these medications to control addiction, but little is known about their efficacy after discharge in this group.

**Objectives:**

The aim is to determine whether the initiation of treatment with specific drugs for alcohol use disorder could help to maintain alcohol abstinence after admission to a General Hospital Psychiatric Ward. In addition, we want to check those factors associated with a higher rate of relapse in consumption.

**Methods:**

We conducted a retrospective cross-sectional study of a group of patients admitted in 2018 to a psychiatric hospitalization ward due to alcohol use disorder and who expressed their desire to stop drinking. At the time of admission, we recorded sociodemographic data, consumption of other substances and alcohol family history. Patients initialized specific treatments to reduce and control alcohol consumption if they wanted. Twenty-four months after discharge, we acquired the number of relapses through new admissions, emergency room visits or outpatient follow-up data.

**Results:**

A sample of 36 patients (28 men) admitted to a psychiatric hospitalization ward was analyzed. At discharge, 17 accepted specific pharmacological treatments to reduce alcohol consumption. After a follow-up period of 24 months, 70.8% relapsed compared to 94.7% who did not accept treatment (χ2=4.001, DF=1, p=0.045, OR=0.13). There were no differences between the two groups in age, gender, amount of alcohol consumed, follow-up modality at discharge or if it was their first detoxification attempt. However, those who did not accept the specific pharmacological treatment consumed other drugs (41.1% vs 5.8%, χ2= 5.888, DF=1, p=0.015), had other history of mental disorder (64.7% vs 23.5%, χ2= 5.845, FD=1, p=0.015) and a higher proportion of relatives with alcohol consumption (81.8% vs 42.8%, χ2= 3.896, FD=1, p=0.048) more frequently. The time (in days) to relapse was faster in this group of patients (200.8 vs 402.7 , Z=-2.5413, p=0.005).

**Conclusions:**

Accepting drug-specific treatment for alcohol use can be helpful for many patients who want to achieve alcohol abstinence. Among the factors that prevent the acceptance of this treatment is the consumption of other substances, comorbidity with another mental pathology and family history, which may involve genetic factors that favour addiction. This group of patients could benefit from a specific pharmacological treatment, although other psychosocial factors may also help.

**Disclosure of Interest:**

None Declared

